# Perceptions of respiratory tract infections and their implications for disease prevention practices among older adults in Mysuru, India

**DOI:** 10.1371/journal.pgph.0004982

**Published:** 2025-07-30

**Authors:** Jantine Marly van Wijlick, K.S. Sahana, B.S. Jayaraj, Christopher Pell, Gangadhar Mysore Rajagopal, P. A. Mahesh

**Affiliations:** 1 Amsterdam Institute for Global Health and Development (AIGHD), Amsterdam, The Netherlands; 2 Amsterdam Institute for Social Science Research (AISSR), Universiteit van Amsterdam, Amsterdam, The Netherlands; 3 Department of Anthropology, University of Mysore, Mysuru, Karnataka, India; 4 Department of Respiratory Medicine, JSS Medical College, JSS Academy of Higher Education & Research (JSS AHER), Mysuru, Karnataka, India,; 5 Global Health, Amsterdam UMC Location AMC, Amsterdam, The Netherlands; 6 Centre for Social Science and Global Health, University of Amsterdam, Amsterdam, The Netherlands; University of Michigan, UNITED STATES OF AMERICA

## Abstract

Respiratory tract infections (RTIs) remain an important cause of mortality and morbidity, particularly among older adults. Annual influenza epidemics cause around 127,000 deaths in India of which 65% are among adults of 65 years and older. Because of India’s rapidly growing older adult population, RTIs are likely to become an even greater public health issue and implementing effective preventive strategies will be crucial. To inform pharmaceutical and non-pharmaceutical prevention strategies for RTIs among older adults, this study explored the perceptions of RTIs and their implications for disease preventive practices of older adults visiting a tertiary hospital in Mysuru, India. Qualitative research methods, including structured observations and in-depth interviews with older adults and healthcare workers were used. Deductive and inductive analysis highlighted how common colds and influenza infections were regarded as inevitable and not considered severe illnesses by older adults. Whereas COVID-19 prompted fear because of severe illness episodes and sudden deaths among family members. Common colds and influenza were often related to eating ‘cold’ food and a ‘cold’ environment, and preventive measures reflected these beliefs. Language played a significant role in the understandings of RTIs in older adults. Different terms for influenza, common cold and vaccination in Kannada and English could lead to lack of awareness of preventive measures including vaccination for RTIs. Because of campaigns during the COVID-19 pandemic, older adults were familiar with non-pharmaceutical prevention techniques such as hand hygiene and social distancing. In conclusion, using relatable medical terms when providing information about RTI prevention and the possibility of taking a holistic approach towards prevention and healthy aging including diet, vaccination and non-pharmaceutical practices means that those are more likely to resonate among the targets. To promote preventive practices, those would ideally also describe the health risks of common RTIs.

## Introduction

Respiratory tract infections (RTIs), caused by various viruses and bacteria, remain an important cause of mortality and morbidity globally [1,2]. Older adults are at particular risk of deleterious health outcomes from RTIs because of structural changes in lung physiology, higher incidence of comorbidities, including hypertension and diabetes, immunosenescence and difficulty in diagnosis [3–5]. Yearly, 60–72% of the deaths due to seasonal influenza, an acute respiratory illness caused by various viral strains, are among people aged 65 and older [6,7]. The recent COVID-19 pandemic spotlighted the vulnerability of older adults to RTIs, with aging as a significant risk factor for severe COVID-19 symptoms, hospitalization, and mortality [8–10].

In India, RTIs among older adults are common. A cohort study with community dwelling adults aged 60 years and older in rural north India found a lower respiratory tract infection (LRTI) incidence of 248 per 1000 person-years and the incidence of LRTI hospitalization was 13 per 1000 person-years [11]. Estimates indicate that annual influenza epidemics cause around 127,000 respiratory and circulatory deaths in India. 65% of the deaths were aged 65 years and older [12]. Reporting on the burden and viral aetiology of RTIs is limited in LMICs, including India, due to incomplete vital records data and insufficient surveillance systems [12–14]. Moreover, little research addresses the prevalence of respiratory viruses during various seasons in India.

India’s older adult population is growing more rapidly than the wider population and with comorbidities, such as diabetes, on the rise, RTIs are likely to become a prominent public health issue in coming years [15]. Moreover, the high levels of air pollutants in India, can lead to chronic respiratory diseases, especially in older adults, thus increasing the risk of severe outcome due to respiratory infection [16–18]. Implementing effective preventive strategies – such as influenza vaccines, which are recommended by the WHO – will be essential. Currently, these vaccines are not part of the National Programme for Health Care of Elderly; with influenza vaccines only available on prescription for a fee at private hospitals, uptake is low among older adults [19,20]. Pre-COVID-19, studies with patients in India revealed limited awareness of infection prevention and control (IPC) practices for respiratory disease [21,22]. In addition, research indicates that solely 1.6% of adults above 60 received an influenza vaccination in 2017–2018 [19]. Vaccine hesitancy, “the delay in acceptance or refusal of vaccination despite availability of vaccination services” [23] and issues with access played a role in the low uptake. It appeared that infrastructure for adult immunization, including influenza was insufficient and that more than 65% of the Indian adults were unaware of adult vaccination [3]. Moreover, many adults had the idea that vaccines are only needed in children [3]. Reasons for non-uptake of the pandemic H1N1 vaccine among Indian adults were costs of the vaccine, lack of access and information, expecting to have a low risk to be infected and a lack of promotion and coercion by the government. Vaccine hesitancy due to lack of confidence seemed less common [3,24]. Motives for receiving the vaccine included awareness of the mortality of pandemic influenza, information from the media and recommendations from healthcare workers (HCWs), friends, or family [24].

India’s response to COVID-19 included a stand-alone vaccination campaign, health promotion and introducing non-pharmaceutical interventions, such as testing, travel restrictions, wearing masks, social distancing, and hand hygiene. Additionally, specific guidelines for adults above the age of 60 and their caregivers were developed [25]. The uptake of COVID-19 vaccines was generally high because of the fear of infection, and upfront- and opportunity cost of hospitalization [26]. Trust in the healthcare system, plus the involvement of Accredited Social Health Activists (ASHA) and local leaders in the vaccination campaign also played a role [26]. The use of non-pharmaceutical interventions was mixed, affected by income and knowledge of COVID-19, and awareness did not always translate to their effective use [27–30].

After the height of the COVID-19 pandemic, little social science research has examined perceptions of common RTIs and disease prevention practices among members of risk groups, including older adults, especially in LMICs. Questions therefore remain about the impact of COVID-19 on efforts to address RTIs and the continued and increasing health threat that they pose to older adults in India. The concepts complacency, convenience, and confidence from the 3C vaccine hesitancy framework developed by the Strategic Advisory Group of Experts on Immunization [23] have a broader relevance beyond vaccination and can be useful to explore perceptions regarding RTIs and non-pharmaceutical prevention practices.

To inform strategies for pharmaceutical and non-pharmaceutical prevention of common RTIs among older adults in India, this study explored the perceptions of RTIs and preventive practices of older adults visiting a tertiary hospital in Mysuru. In addition, this study aimed to understand the implications of the health system context including the interaction with healthcare workers (HCWs), such as nurses, doctors, pharmacists, and therapists, for the RTIs preventive practices of older adults.

## Materials and methods

### Study design

This research draws upon qualitative methods including, in-depth semi-structured interviews, structured observations, and informal conversations with older adults, their family members and HCWs. Applying multiple research methods and including different stakeholder groups enables triangulation of the findings, reduces risk of bias, and improves validity [31].

### Setting

The data collection took place at a tertiary teaching hospital and urban health centre in the city of Mysuru, located in Karnataka state in southern India. Interviews and observations were conducted from 2^nd^ of January 2023–12^th^ of May 2023. The hospital provides care to approximately 27,000 outpatients each month and can have 1800 patients admitted [32]. The patients live in the Mysuru, Chamarajanagar, Mandya, Coorg, and Hassan districts of Karnataka and the Nilgiris district of Tamil Nadu [32].

### Sampling and respondent recruitment

The study aimed to include a diverse research population of HCWs and older adults in terms of age, gender, and educational level. Older adults, commonly termed “senior citizens” aged approximately 60 years and older were purposively sampled and invited for an interview. The age of 60 + corresponds with the definition of an older person applied by the United Nations [33]. Older adults who were not able to give consent with or without the help of a family member, who had moderate to severe dementia and inadequate ability to speak English or Kannada were excluded. If they were interested and made the respondent feel more comfortable, their accompanying family members were also present during interviews. The older adults were in- or outpatients at the respiratory medicine, general medicine, geriatrics departments of the hospital or were patients at the urban health centre. The patients and their family members were invited to participate by the research assistant during a hospital visit or by the staff from the urban health centre.

Healthcare workers, including junior to senior level nurses, doctors, therapists, and pharmacists from the respiratory medicine, general medicine, geriatrics outpatient or in-patient departments of the hospital, the urban health centre, and the medical pharmacy, who inform older adults about the influenza vaccine or who are otherwise involved in the influenza vaccination purchasing-supply chain, were purposively sampled and invited to participate. The HCWs were contacted via telephone or email or approached in the hospital. Recruitment continued until theoretical saturation had been reached [31]. The older adult participants received some fresh fruits and the HCWs a notebook and a pen to thank them for their participation.

### Data collection tools

The interview guides (S1 Appendix and S2 Appendix) were based on the 3Cs and the vaccine hesitancy determinants matrix developed by MacDonald et al. [23] and were adjusted when additional relevant topics came up during the interviews. Vaccine hesitancy is defined as “the delay in acceptance or refusal of vaccination despite availability of vaccination services” [23]. The 3Cs of the vaccine hesitancy framework consist of three factors including confidence, complacency, and convenience. The vaccine hesitancy determinants matrix includes factors that can influence the decision to discard, postpone, or accept (specific) vaccines divided into the following three categories: contextual influences, individual and group influences, and vaccine/vaccination-specific issues [23]. The 3Cs and the factors from the matrix have broader relevance beyond vaccination and were found to be helpful for exploring perceptions regarding RTIs and non-pharmaceutical prevention. For instance, complacency includes risk perception of the infectious disease, convenience includes health literacy and ability to understand preventive services and part of the contextual influences are beliefs and attitudes about health and prevention [23]. Prior to conducting the interviews, the researchers discussed the guide to ensure a shared understanding of the questions, to improve internal validity.

### Data collection

A Dutch PhD researcher (JvW), who has experience in qualitative research methods, and an Indian research assistant (SKS), who obtained a MSc in anthropology and has experience in qualitative interviews, conducted in-depth semi-structured interviews with older adults, their family members and HCWs. When the participants preferred to speak Kannada, the mother-tongue for most of the population in Karnataka, the research assistant acted as an interpreter. The semi-structured interview method allowed the researcher and assistant for probing and allowed the participants to use their own vocabulary and to choose the order of the topics [31]. The interviews took place in-person in the hospital, urban health centre or online via Microsoft Teams or Zoom and had a duration of 15 minutes to 75 minutes. Depending on the preference of the respondent and on the ability of the researcher to travel to the hospital or urban health centre the interviews were conducted in-person or online.

To complement the in-depth interviews, structured observations, and informal interviews with HCWs, older adults and family members were conducted. Those took place at the respiratory medicine, general medicine, geriatrics outpatient or inpatient departments, the medical pharmacy, drug store, intensive care unit, the special ward, paediatrics outpatient department in the hospital and the community medicine department of the hospital’s medical college. These structured observations enabled the researcher and the research assistant to familiarize themselves with the social context of the hospital and the urban health centre, and to have informal conversations with the respondents to get additional insights about older adults’ perceptions regarding RTIs and their implications for RTI preventive practices.

### Data processing and analysis

Once approved by the participant, the interview was audio recorded, allowing for detailed transcription and translation from Kannada to English. Besides, during the interview, notes were taken, which were useful for rapid adjustment of the interview guide prior to full analysis and when the recording quality was poor. Field notes were written during and after the structured observations. Furthermore, a rigorous process of going back and forth through the data was applied by the researcher and research assistant to understand the meanings of Kannada and English terms regarding common infectious diseases and preventive methods used by the participants.

The transcripts and fieldnotes were analysed in MAXQDA 24 [34] by JvW. First deductive coding was applied, based on the 3Cs and the vaccine hesitancy determinants matrix from Macdonald et al. [23]. Then, inductive coding followed which was data-driven: additional or alternative codes and themes were developed directly from the data. In February 2024, feedback was received from HCWs from various departments of the hospital and the urban health centre during a dissemination visit by JvW and SKS. The COREQ checklist (S3 Appendix) has been used for reporting the methods and results [35].

### Ethical approvals and informed consent

Approval from the Institutional Ethics Committee of the JSS Medical College in Mysuru, India has been obtained on 15 October 2022 (Project filed as: JSSMC/IEC/101022/ 32 NCT/ 2022–23). Additionally, approval from the Ethics Review Board of the Faculty of Social and Behavioural Sciences, University of Amsterdam, The Netherlands has been given on February 22nd, 2022 (Project filed as: 2022-AISSR-14382). Prior to the informal conversations and interviews, the potential respondent received an information flyer, which provided an introduction of the researcher and assistant including their contact details and name of the university in Mysuru, an explanation of the purpose and procedure of the study, that their participation will be voluntary and that they can withdraw from the study at any moment without consequences. Additionally, it was mentioned that the collected data would be anonymized and safely stored. The information flyers were written in basic English or Kannada. Moreover, the font size was enhanced to increase readability for older adults with visual impairment. In addition, the reason for the research topic was explained to the participants.

Then, depending on the setting, verbal or written informed consent from the respondent was requested for the interview and its recording. Verbal consent was not recorded but registered in a document on an encrypted laptop and on a save drive. During the interviews consent to continue the conversation was asked several times which is in particular important when studying vulnerable older individuals [36].

## Results

From January to May 2023, respectively 24 and 26 semi-structured in-depth interviews were conducted with older adult patients and HCWs in the hospital and the urban health centre in Mysuru. Four interviews were initiated in a more informal way and were therefore not recorded but detailed notes were taken during and after the interviews. In addition, structured observations, which involved informal conversations with 40 HCWs and 14 older adult patients took place between January and May 2023. 15 family members of the patients joined parts of the interviews and conversations to support the patient. Five older adults refused to participate because of feeling too unwell to speak or insufficient time due to an upcoming appointment. 10 HCWs refused participation because of being too busy or not wanting to participate in an online interview.

Table 1 presents the demographic details of the older patients who participated in the semi-structured in-depth interviews. Most patients were between the age of 58 and 78 and most were female. The youngest patient was 57 years old, and the oldest patient was 90 years old. One third of the participants worked in agriculture. More than fifty percent of the patients did not finish primary school or did not receive any formal education. 14 participants lived in a village or town and the rest in the city of Mysuru, often living with at least one family member. Most interviewed older adults were patients in the geriatrics department and were out-patients. In the interviews, participants spoke Kannada except for one interview which was conducted in English.

**Table 1:** Characteristics of participating older adults.

Total in-depth interviews with patients	24
**Age group in years**	
38–58	1
58–78	14
78–98	5
unknown	4
**Gender**	
Male	10
Female	14
**Last profession (before retirement)**	
Agricultural worker	6
Silkworm/plastic factory worker	3
Childcare/nurse	2
Hotel/restaurant manager	2
Incense maker	2
School/Mosque teacher	2
Cleaner	1
Construction worker	1
Factory owner	1
Lab worker	1
Tailor	1
Technician	1
Never worked	1
**Educational level (highest finished)**	
Never went to school	8
Primary school (Standards I to VIII)	4
Secondary School (Standards X-XII)	7
Vocational education	2
Undergraduate (Bachelor)	1
Graduate (Master, Post-graduate, etc.)	0
Unknown	2
**Place of living**	
Village	11
Town	3
City	10
**Living situation**	
With 1 family member	8
With more than one family member	10
Alone	1
Unknown	5
**Department**	
Geriatrics	10
Respiratory medicine	6
Urban Health Centre	7
**In/outpatient**	
Out-patient	15
In-patient	9

Table 2 includes the characteristics of the interviewed HCWs. Most were between the age of 18 and 38 and were female. More than two thirds of the HCWs were medical doctors, junior or senior and two third worked at the respiratory medicine department or geriatrics department. In 17 interviews the HCWs spoke English and in eight interviews they spoke Kannada.

**Table 2:** Characteristics of participating HCWs.

Total HCWs in in-depth interviews	26
**Age group**	
18–38	20
38–58	4
58–78	2
**Gender**	
Male	7
Female	19
**Medical profession**	
Physician	17
Nurse	5
Pharmacist	1
Occupational therapist	1
Community/public health professional	2
**Department**	
Geriatrics	6
Respiratory medicine	10
Medical pharmacy	1
General medicine	4
Urban health centre	5

### Perceptions of common respiratory infections

#### Terms, symptoms, and severity.

Older adults used various terms, in Kannada and English, to describe common respiratory infections, including influenza and the common cold. When talking about the symptoms of common respiratory infections, respondents described throat pain, cough, chills, fever, and diarrhoea. They mentioned diseases such as ‘*śītajvara’* (ಶೀತಜ್ವರ)*,* which literally means ‘cold fever,’ or ‘*śīta,’* which are both used to refer to influenza. Many had heard of ‘flu,’ ‘flu fever’ or ‘*śīta(jvara)*’** and the symptoms that the older adults described were fairly similar. Frequently mentioned symptoms included fever or increased body heat, cold, ‘chills,’ cough, and headache. In addition, body pain, a blocked or runny nose, throat itching, mucus, loss of appetite and sneezing were reported.

*I: Did you hear about flu?*

*P: Yes, but I don’t know much about that*

*I: Did you hear of śītajvara?*

*P: Yes […] Cold may be a runny nose or mucus which blocks the nose. The runny nose happens due to increased body heat. Throat itching also happens. […] Cough will occur due to flu as well […] And even mucus will come out when we have flu.*

Interview with a 62-year-old woman (P26)

Besides being seen as a symptom of ‘flu’ (the term flu will be used in this paper because the term influenza was not used by the older adults), ‘cold’ was also seen as a result of the flu or as a separate illness.

*P: If we get chill fever, flu will increase. Cold is more [dangerous] than flu.*

*I: How does cold happen?*

*P: A runny nose, the head feels heavy and throat itching. [..] Then [when we have a cold] we will go to the doctor.*

Interview with a ≈ 70-year-old woman (P22)

Several adults explained that flu leads to ‘cold’ (referred to as common cold in this article, to distinguish between cold as an illness and cold as a bodily experience), which is more severe. Some said that common cold is less severe than flu while others said that both conditions give the same symptoms. A few HCWs had difficulties differentiating between flu and common cold as well. The Kannada term ‘*negaḍi’* (ನೆಗಡಿ) was also used to describe the common colds. Some older adults also talked about ‘cold and cough’ as a specific infection which seems similar to flu and the common cold.

When talking with clients about flu, besides the English term, a senior physician said she would use the Kannada terms ‘*viral jvara’* (ಜ್ವರ) which literally means ‘viral fever’ and ‘*handi jvara’* (ಹಂದಿ ಜ್ವರ) which means swine flu or H1N1:

*‘[Patients] understand [flu] in Kannada. We use the word of viral jvara. So, they understand those terms. [...] They know that in winter they get jvara. “Viral jvara” they will say. And everybody knows a term called handi jvara*.’

Interview with a senior physician (H44)

For many older adults, flu was not considered a severe disease, and they were not too worried because they had either suffered from it several decades ago, never experienced it, or had contracted it recently but did not get very sick from it. It was not a topic of conversation among family members or friends.

*‘Many relatives of ours died. [...] People used to talk about the covid death cases (and) that’s why [many relatives] died. [...] They died due to the fear of corona, if they got fever, they used to think we got corona and due to that fear, they used to die the very next day.’*

Interview with a 72-year-old man and his wife (P2 & F2)

Fear of COVID-19 played a significant role in the lives of the older adults during the height of the pandemic. Even though COVID-19 infections (frequently termed as ‘covid’ or ‘corona’ by the older adult participants*)* often led to similar symptoms to those of common cold and flu, COVID-19 was seen as a more dangerous condition. In contrast to the flu and common cold, participants highlighted how neighbours and family members would die within several days due to COVID-19 and mentioned severe and long-lasting breathing problems. At the time of the data collection, participants said that COVID-19 was not ‘on their mind’ anymore, and when people have flu-like symptoms, they no longer immediately associate those with COVID-19.

#### Causes.

That certain diseases can be contagious was mentioned by several participants. Older adults reported that flu can spread from one person to the other. Tuberculosis (TB), cholera and especially COVID-19 were also mentioned. COVID-19 was seen as a highly contagious disease that spreads very fast, ‘like wildfire’. Some recognized a virus as the cause of common respiratory infections. Although one respondent explicitly questioned that respiratory diseases could be contagious, many more older adults saw common respiratory infections as also related to certain environmental factors and diet.

*‘[Cold, cough and fever] will come and go. If we work in a cold environment and if we get wet in the rain then we will get cold, cough. But now since it is summer, I don’t have any problem.’*

Interview with a 62-year-old woman (P18)

As this respondent explained, people often assume that their common cold or flu infection resulted from environmental factors, including being soaked by rain and bathing, working, or playing in cold water or a cold environment. One HCW also explained the cause of her fever or throat infection in terms of cold weather rather than infected patients in the hospital. Also, poor quality air and fog were mentioned as causes for flu, common cold, cough and breathing problems. In addition, symptoms of respiratory infections were described as more frequent during wintertime and *cold* or rainy season. Given the environmental aetiology, respondents described how anyone is at risk of common cold and flu.

Furthermore, according to some, smoke, pollution, poverty and living in slums can cause respiratory infections due to an unclean environment and unhealthy diet, such as eating tobacco and drinking alcohol:

*‘Smokers and alcoholics are seen as TB patients. When we won’t eat proper food, consume alcohol, and smoke cigarettes and also tea, it will lead to TB.’*

Interview with a ≈ 70-year-old woman (P25)

Connections between common cold, feeling cold and ‘cold food’ and drinks were also made by respondents. Food that could cause common cold and flu were described as curd (homemade Indian yogurt); certain fruits, such as guava, cucumber, and banana; ice cream and cold water. Although not per se cold in terms of temperature, as the older adults explained, these food items give a colder bodily sensation and are therefore classified as ‘cold foods’. Other dietary changes, such as changing the type of drinking water, was also linked to respiratory infections.

### Ideas about prevention and cure

Respondents mentioned extensive health promotion campaigns regarding diminishing the spread of the coronavirus during the COVID-19 pandemic. The older adults described using a respirator during the pandemic because this was recommended to them, for instance by HCWs at the vaccination centre and by their doctor. They were told that the mask prevents infection and inhalation of polluted air, which was in particular important if they had experienced severe COVID-19 already. At the time of data collection mask wearing was less common.

*P: […] Covid made us wear a mask. At that time [of the covid pandemic], we were not going anywhere. Even if someone died, we didn’t go out to visit that person. […] This [knowledge about prevention] is due to publicity. Because it is widespread, everyone knows about it. And the police came and announced it like: ‘don’t sit outside, wear a mask, don’t gather in groups, don’t go anywhere, don’t spit on the road.’ And when we speak to others or cough, [we] wear a mask. And if anyone has common cold and fever, they put everyone in a corona room. Because of that so many members died. [...] One of our relatives got it. At that time, I went and clean their house. I didn’t get anything. But we can’t avoid getting infections.*

Interview with a 62-year-old woman (P26)

Hygienic measures, such as washing hands and keeping distance from people with symptoms, such as coughing, were seen as important for some to prevent COVID-19, flu and common cold infection. However, the older adults made it clear that following hygienic measures and keeping distance was not always possible in the case of taking care of a family member and when staying at a busy ward. Besides, getting treatment in isolation at the hospital is too expensive for many people means that they often recover at home. A few older adults said that decades ago, during previous flu, ‘plague’ and cholera outbreaks, people would flee their village out of concern about potential infection. Another traditional preventive method was the ‘nose prick,’ a religious ritual which was used by the parents of an older adult to prevent him from dying from flu, plague, and cholera.

Many interviewed HCWs confirmed that they educate patients regarding non-pharmaceutical prevention methods of common infectious respiratory diseases. They advised their patients, and in some cases the person accompanying the patient, to wear a mask, to cover their mouth while coughing and explained them about hand hygiene. Some recommended patients to maintain distance and to isolate themselves at home when admitting them was either not possible or not needed because they were not infected with COVID-19, H1N1 or TB. In the hospital, non-pharmaceutical preventive advice was often provided when the patient was infected and was therefore mainly about preventing transmission to others. According to some HCWs, the health education to patients regarding non-pharmaceutical RTI prevention increased during the COVID-19 pandemic, whereas for others, especially at the respiratory medicine department and urban health centre it was already common practice to educate patients and the community about common seasonal infections and TB prevention, where to receive treatment and how to minimize transmission. The HCWs at the urban health centre organized community awareness programs and would go house to house to explain to the community about TB prevention. In addition, for any communicable disease controlling the outbreak is especially important to them. Two HCWs said it can be more challenging to convince older adults about the importance of preventive measures and that developing trust is particularly important:

*‘First, they should trust us. […] This is not a one-day process. I am working here since five years, I visit the people often and I will get to know them. We need to develop rapport with them. And then we should make people feel like, they will get some health benefit from us. We should behave with patience, then only we can achieve something. […] Here there are 2050 houses, each and every house is important. A single house can spread the infection to the entire area. […] When it comes above 60, they are bit lonely and don’t have support from children. So more than a medicine they need concern. If we just talk to them, they feel happy. They won’t approach us first; we should approach them first.’*

Interview with a public health professional (H56)

The environmental and dietary understandings on causation of common respiratory infectious illnesses influenced the ways older adults described preventing these illnesses. There were several foods that should be avoided to prevent flu and common cold infection according to them. For instance, to prevent getting sick with the flu or ‘*śītajvara’*, oily and spicy food and fruits including apple and banana should be avoided. Three older adults recommended not eating ‘outside food,’ (non-home cooked meals). They advised drinking solely hot water, warm or liquid food and beverages.

For a cold and cough, consuming certain preparations of pepper and chili were seen as preventive. In addition, ‘*ragi* ball,’ which is made from finger millet and water, was suggested by several older adults and a family member. There were some contradictory recommendations as well. For example, even though according to some eating cucumber causes flu and should be avoided, several older adults said that cucumber can cure a cold and cough.

Some older adults said that the dietary restrictions only need to be followed in the rainy, cold season or winter. For some, preventing common respiratory infections was seen as impossible because their causes – cold weather, rain, and ‘cold foods’ – cannot always be avoided. Four older adults said that it is normal that everyone gets sick once in a while.

The dietary precepts were a result of personal experience, or from neighbours and healthcare providers. Eating healthy and home cooked food, including eggs, fruit, and leafy vegetables, was seen as important to avoid infectious diseases, such as TB and flu because they improve ‘immunity power’ according to several HCWs. One HCW highlighted that having a full stomach is important especially at an older age because of immunosenescence.

### Perceptions of vaccination

#### Language.

Several factors influenced the older adults’ perceptions of adult vaccination. In Kannada, vaccination is translated as ‘*lasike’* (ಲಸಿಕೆ) and according to some older adults, vaccination or *lasike* is only given to children, to protect them against polio for instance. Several older adults noted that they did not get any ‘*lasike’* as a child, but others mentioned receiving a childhood vaccination against smallpox. Certain ‘marks’ on their skin were sometimes related to smallpox or childhood vaccinations including BCG vaccination. A minority knew about the flu vaccination for children, but not for adults.

Frequently older adults reported never having received a vaccination or ‘*lasike.’* Many, however, described having had the ‘covid injection,’ which some understood as preventive, but none could explain how it worked. Not experiencing side effected was itself seen as positive.

*I: Do you know what is vaccination (English term)?*

*P: No, I don’t know.*

*I: Did you hear about lasike?*

*P: Yes, it’s given to children. […]*

*I: Did you ever take lasike?*

*P: No […] I know it only helps children. I learned it from the doctor. And our grandchildren are getting it. But we never got it, because in our time there were no such facilities.*

Interview with 65-year-old woman (P24)

Although most older adults reported not receiving any other vaccination during their adulthood, a few said that they had received the one-year *(‘ondu varshada’*/ ಒಂದು ವರ್ಶದ*)* injection. Which, according to the HCWs, is the seasonal flu vaccination. One patient at the respiratory medicine department received the ‘one-year injection’ for nine years in a row, except for one year during the COVID-19 pandemic. She also received the ‘five-year injection’ (probably the pneumococcal vaccine), for the first time during the same year of the data collection.

Some doctors and nurses said that when they would explain about adult flu vaccination to their adult patients, they would use the English term ‘one-year-injection’ or the Kannada translation ‘*ondu varshada injection’* to communicate this to their patients and specify that it was the annual vaccination. According to them this word was also useful to find out whether the patient had been vaccinated in that year already.

For some HCWs, language barriers made explaining preventive measures to patients challenging. This was particularly the case for the post-graduate physicians and interns in the hospital who are not from Karnataka and therefore do not speak Kannada. The interviewed HCWs reported that this is mainly a problem when addressing older adult patients because they often do not speak English. A doctor suggested that nurses who are often fluent in Kannada should be responsible for explaining about adult vaccination.

#### Trust and safety.

The media, including TV news channels and (community) HCWs played a role in spreading information about COVID-19 vaccination to older adults. In addition, everyone would get text messages from the government about the COVID-19 vaccines and information about the vaccine would spread from person to person in communities and families. Besides, HCWs would tell everyone in the neighbourhood or village to get vaccinated. However, even though people got information about the COVID-19 vaccine from various sources, there was distrust, especially in the early stages of the pandemic. Stories about side-effects and deaths linked to COVID-19 vaccines spread in the community. Although most older adults only experienced minor side effects, such as pain on the injection site and a few days of fever, three older adults experienced long-term complaints, which they associated with the COVID-19 vaccination. They experienced backpain, fungal infection and joint pain and were not eager to be vaccinated against flu. Another reason for hesitancy about the vaccine was assumptions that it would reduce the effectiveness of her body’s immune response:

*‘When people take vaccines, you’re killing your immunity. I rather get properly immune, naturally, then taking a vaccine and getting immune. That is my feeling. [...] If we are in contact with people that have the influenza, we rather stay away then taking the vaccine, isn’t it?’*

Interview with a 71-year-old woman (P12)

After some initial hesitancy, most older adults were vaccinated and saw the COVID-19 vaccine as effective because they had either minor complaints or did not get COVID-19 after the vaccine. Only a few said that you can also protect others by getting vaccinated.

According to HCWs, among older adults, distrust of adult flu vaccination was not common. However, it was not seen as a priority by many:

*‘Yes, you have to convince the patient about vaccinations. […] If it is not a therapeutic thing then they won’t generally do that.’*

Interview with a post-graduate physician (H6)

Healthcare workers described how preventing disease is seen as less of a priority among patients, especially according to the elderly. Some older adults themselves indicated that they are ‘ready to die’ and therefore do not see the need of getting vaccinated against the flu. Certain HCWs and departments seem to be more inclined to focus on treatment than vaccination. Most older patients said that they have never talked with their physician about adult vaccination. This contradicts what several HCWs said at the pulmonology, geriatrics, and general medicine. They highlighted that they do recommend the pneumococcal and flu vaccine to their patients, especially to older adults and when they have comorbidities. Thus, recommendations varied between HCWs in terms of who to recommend it to and when.

*‘If you look outside our field, very few other departments recommend regular vaccination for people with severe comorbidities, such as severe impairment of lung function […]. They [colleague physicians] are more worried about treating the current state and the current disease rather than thinking about the prevention. So, I think another way to improve vaccination would be to […] educate us doctors more and ask us to recommend vaccination to the patients much more.’*

Interview with post-graduate physician (H8)

The nurses and physicians reported that they became aware of adult influenza vaccination once they started working at departments where such vaccination is recommended to adult patients. A postgraduate doctor said that her supervisor advised her to read more about influenza vaccination for adults however she did not dedicate time for it. Several HCWs explained how the COVID-19 and H1N1 pandemics increased the awareness of adult influenza vaccination among themselves and community members.

Recommendations regarding vaccination from trusted HCWs were seen as important by the older adults. Besides, several older adults said that they would only take the flu injection if other people would accept it and have no complaints after the vaccination:

*‘Yes, I will take it. But other people should also take it, and they should tell it doesn’t give any problem. Then I will ask at home and then I will take it. But if they tell the injection is provided only to people who have flu, not to people who don’t have it, I won’t take it.’*

Interview with a 70-year-old woman (P27)

In the hospital there was no policy regarding vaccination for older adults. This contrasts with the COVID-19 vaccination campaign, which incorporated outreach campaigns led by healthcare providers. In contrast to the flu vaccination, compulsion by the government was a reason for getting vaccinated against COVID-19. According to some older adults, without a COVID-19 vaccination they could not keep their ration card, which is used by people with a lower socio-economic status to purchase subsidised food. Moreover, travelling across the border of different Indian states or travelling by train was restricted without a proof of COVID-19 vaccination. For the latter reason two older adults still decided to get vaccinated even though they were worried about the side effects of the vaccine.

### Vaccine access

Several barriers regarding adult vaccination access were pointed out especially by the HCWs. Except for the COVID-19 vaccination, adult vaccination is not provided for free. Healthcare workers said that for some the vaccine is too expensive. As some interviewed older adults mentioned, they are dependent on their family members in terms of transport and also financially, which according to some HCWs in combination with not finding preventive measures important, makes them refuse flu vaccination or postpone it.

*‘The burden here comes with money. So, for them, spending thousand five hundred, six hundred on a vaccine is kind of, they are not inclined to get vaccines [...] to prevent an infection by spending so much. [...] If something is made free [...] we could influence more people to get it done. [...] Our elderly population, most of them are financially dependent on someone else. So, they are dependent on their children […] Many times, they are not open to getting it. And they will probably say that they are scared about getting it done. But many times, it would be the monetary thing which actually becomes the factor for them not to get it.’*

Interview with a senior physician (H45)

Other HCWs said that, in their experience, patients with recurrent RTIs are more eager to accept flu vaccination, particularly when they explain to them that the costs of being admitted to the hospital are higher than the costs of a yearly flu vaccination. A patient at the respiratory ward confirmed that even though the vaccine is expensive that because of her health condition she should get the ‘one-year vaccination’.

Outside of the hospital, it seems more difficult to receive adult vaccination. A patient who receives the flu vaccination yearly explained that in the hospital the ‘one-year injection’ is always available but because no nurse was available it was not possible to be vaccinated in her own village. Furthermore, at the urban health centre adult vaccinations were not available. Even though infectious disease prevention, including TB, are an important part of their work, none of the interviewed HCWs and older adults at the urban health centre were aware of the possibility for older adults to get vaccinated against flu in the main hospital.

Although the COVID-19 vaccination was electronically registered, the HCWs explained that the flu vaccine is solely registered in a patients OPD card when they are being discharged or visit the OPD. Besides, no invitation system exists for the flu vaccine. Mainly at the respiratory medicine department the doctors said that because of this there is a problem with follow-up. They recommend the vaccine, and a patient receives it on the spot, but if this is not a regular patient, then often the patient does not come back next year for the vaccination. Or the patient explains that they first need to save money for the vaccine but in the end do not return.

### Treatments

In addition to plain hot water, ‘*kashaya,’* an ayurvedic herbal drink, was seen as a way to treat and prevent common respiratory infections. The drink often consists of a combination of spices and jaggary in hot water. It is frequently used to diminish symptoms of a common cold or flu, mild throat infection, cough, throat itching, removes mucus and is supposed to keep the body warm. Furthermore, it has been used during the pandemic to prevent COVID-19. Sometimes this drink was recommended by doctors but often the recipe has been spread by word of mouth:

*‘Tulsi, pan leaf, cumin seed, pepper, and honey. We will boil it with water and along with that we will also add jaggary or sugar, then we will drink it. [...] People will tell us if we prepare and drink this kind of Kashaya we will get cured. So, we will try the recipe given by them and we tell others who don’t know about this. Like this information will pass from person to person.’*

Interview with a ≈ 70-year-old woman (P22)

In addition, steaming – either with or without certain herbs or spices – was a common flu treatment used by older adults. Warm foods, including porridge, were mentioned. Choosing a certain diet did sometimes also depend on the costs:

*‘Ragi flour is filtered using a cotton cloth. Then white flour is separated from black flour. From the white flour we will make porridge and drink it. [...] Bread, coffee, and biscuit. Sometimes fruits like orange. If we have money, we will buy all these. If not, I will eat porridge.’*

Interview with a 60-year-old woman (P19)

A HCW at the urban health centre explained that they give dietary recommendations to the patients who have flu-like symptoms, such as drinking ‘*Kashaya’* and consuming liquid and easily digestible food which is part of ayurvedic medicine and passed from generation to generation. Most HCWs talked mainly about applying these home remedies for treating themselves and their family sometimes combined with steaming, saltwater gargling, and allopathic medicine, which is available at the local pharmacy.

Home medication remedies were often combined with allopathic medication. If symptoms worsened, respondents described visiting their local pharmacy for ‘tablets’ against cough and cold or *‘Dolo’* (paracetamol) and antibiotics. Most reached out to their doctor when symptoms worsened. In such cases they might receive tablets, sometimes injections and treatment in the hospital. However, treatment in the hospital was often seen as expensive, time consuming and, for some, because of their age, it was difficult to reach the hospital. Two family members explained that it is therefore more common to first receive treatment at a private clinic. During the COVID-19 pandemic people would visit the hospital even less out of fear to be isolated or anxiousness regarding the treatment for COVID-19 according to an older adult. Several older adults said that they choose to not take ‘home medication’ but follow their doctors’ recommendations instead.

## Discussion

This study highlighted how at the final months of the COVID-19 pandemic period, among older adults visiting a teaching hospital in Mysuru, India, there remains significant distinction between attitudes towards COVID-19 and other respiratory infections – some of which have implications for prevention efforts. Understandings of aetiology and approach to preventing influenza, the common cold and other respiratory infections were quite different to those related to COVID-19. To a certain extent, influenza and common cold were seen as inevitable – linked to environmental conditions – and prevention was related to diet, with some consideration for non-pharmaceutical interventions. However, for most older adults, COVID-19 was a much more severe disease that spreads very fast compared to influenza or the common cold. This seemingly led to a greater risk perception, which influenced attitudes towards and uptake of vaccination and other non-pharmaceutical preventive measures. These insights in perceptions of seasonal respiratory infections, the pandemic COVID-19 virus and applied preventive practices could be valuable for the implementation of preventive methods for endemic and emerging respiratory infectious diseases by (local) policy makers.

Through visible and impactful public health campaigns, the older adults in this study had become familiar with the symptoms of and preventive interventions for COVID-19. As part of the response to the pandemic, and with awareness for their exacerbated vulnerability, HCWs and local authorities instructed older adults to strictly follow guidelines, such as wearing a mask and social distancing. In India as a whole, especially during periods of lockdown, when population movements were restricted, the speed of the epidemic decreased, with a growth rate for confirmed cases from 21% on 30^th^ of January to 6% on 4^th^ of May 2020 [28]. Following the guidelines was, however, not always possible. For example, keeping distance from sick family members was difficult, particularly in crowded urban housing [37]. Moreover, as part of the COVID-19 response, restrictions were also placed on travel and according to the older adults, access to food support by the government with a ration card for the unvaccinated was denied. These restrictions were cited as motivating several hesitant older adults to get vaccinated. The motivation to get vaccinated because of travel restrictions is consistent with findings from surveys from the United Arab Emirates and Spain [38,39]. Similar to this study, other research in India found that compulsion by the government was a reason for respondents to get vaccinated, however, hindered access to food support was not mentioned [40], and appears to be a rumour spread via social media [41] and (community-based) HCWs. No such motivation was seen for other adult vaccinations which were seen as costly and not needed. Lack of access to vaccination due to opportunity costs, which the family of the older adult has to meet, was a reason for older adults to either postpone or reject vaccination according to HCWs. Access issues because of the cost of the vaccine have been described by studies in other LMICs [42,43].

Among the older adults, the COVID-19 pandemic and the public health response had a relatively minor impact on perceptions of other RTIs and their preventive measures, including vaccination. Even though awareness regarding RTIs increased, traditional hot-and-cold beliefs and practices stayed intact. Possibly, because pandemic diseases such as H1N1 and COVID-19 are viewed as a different category from seasonal diseases due to their great impact. In addition, for centuries, other systems of healing have existed alongside and contributed to allopathic medicine [44]. Many older adults saw being exposed to cold or rain as a cause of common cold and influenza. This reflects epidemiological studies that, besides round-the-year circulation, show biannual peaks of influenza during winter and monsoon season in various regions in India [45–47]. Similar seasonal circulation has been observed for other respiratory viruses, including human rhinovirus which is causing common cold [48]. With this environmental causation, bouts of RTIs were seen almost as inevitable. Consuming food said to cause ‘cold to the body’ was seen as a cause for common cold and influenza. Hence, eating home cooked food and drinking hot water often mixed with certain spices would contribute to increased immunity and diminish severity of disease. Especially drinking hot water with spices is used as preventive and curative measure against infections in Ayurveda, the traditional medicine system, which originated in the Vedic period in India [49,50]. Moreover, anthropological studies on hot-cold concepts found that these concepts are applied for understanding, treating, or preventing disease and infections world-wide across high-, middle, and low-income countries [51,52]. Greater attention from HCWs to the hot- and cold concepts that older adults apply to prevention and treatment of RTIs by including this in medical education programs for HCWs could be beneficial. Future research could focus on how to connect these concepts with preventive interventions for older adults.

Diet has been linked with inflammation, which affects the risk of chronic disease and complications linked to infections. Poor nutritional status could predispose individuals to infection, and it could lead to an aggravated immune response towards an infection [53]. Besides, obesity and associated comorbidities are related to psychological changes, which leads to rapid virus shedding, higher susceptibility to infection and pathogenicity of respiratory infections, such as COVID-19 [53,54]. In addition, perceived vulnerability of infection and the severity of the disease is one main motivator influenza vaccination uptake among high-risk groups in LMICs [43]. This demonstrates a need for HCWs to emphasize the potential risk of RTI for older adults and patients with comorbidities to increase the uptake of adult vaccination and adoption of non-pharmaceutical interventions. Moreover, a more holistic approach towards prevention and healthy aging including a combination of non-pharmaceutical prevention, vaccination, and a healthy diet, which was highlighted by the older adults, is suggested to improve the immune response and to reduce the risk of severe outcomes due to obesity and diabetes. This could be promoted by senior doctors and included in trainings for HCWs.

In contrast to the awareness of COVID-19 vaccination, most respondents did not know about any other adult vaccinations. Similar to studies about adult vaccination in LMICs [43], the older adults experienced a lack of recommendations from HCWs. The use of language appeared to be important for health communication regarding prevention. The finding that older adults mainly knew COVID-19 vaccination as an injection or ‘*lasike’* and did not see child immunisation as a similar preventive concept is an important contribution of this study regarding understandings of vaccination. Only a few older adults had been vaccinated against influenza, which they would refer to as one-year injection, and most older adults had never heard of influenza vaccination before. Healthcare workers would often use a combination of Kannada and English words to communicate with their patients about infections and vaccination. A cross-sectional survey study at emergency departments in India showed that because of the low health literacy of some patients they have to translate medical terms to the local language, which is challenging, especially if there are no analogous words for certain medical terms [55]. Besides, some HCWs did not speak the local language, which was especially an issue for older adults, from rural areas and with a lower income, who often do not speak English [56]. Not being proficient in the same language can be difficult when explaining about a disease and especially prevention such as vaccination. A longitudinal study from India found that the social proximity, including sharing a common language, plays a significant role in TB knowledge and infection prevention behaviour [21]. Because older adults are often dependent on their younger family members who might be more proficient in English, this might lead to these younger family members deciding what preventive measures will be followed. Thus, when informing older adults about preventive methods, awareness of medical terms being used is relevant. Therefore, training on culturally appropriate communication for HCWs could be beneficial. Even though more people in India are able to speak English, understanding terms regarding disease and for instance vaccination can still be a challenge. Especially if there is no literal translation in the local language for these terms.

Research from European countries has shown that besides limited language skills and health literacy, limited digital skills and misinformation from social media, friends and family are an important reason for a lower COVID-19 vaccination uptake among citizens with a migration background, people with a lower education level and older adults [57,58]. In addition, studies from India about vaccine hesitancy among parents regarding children’s vaccines and among older adults regarding COVID-19 vaccines, stated that these groups often rely on information about vaccination and the pandemic from social media, such as WhatsApp, YouTube and Instagram, instead of reliable sources, governmental websites or local HCWs. In the present study, older adults however did not explicitly refer to social media as a source of information on infectious diseases and vaccines [59–61]. Because older adults’ knowledge about the existence and the access of adult vaccination is limited except for COVID-19 vaccination and because older adults often find traditional methods of prevention and cure of RTIs effective, this could be the reason why social media was not mentioned by them as an important source of information. In addition, this could be a reason why concerns regarding safety and effectiveness of adult vaccination were not often mentioned in contrast with other vaccination acceptance studies in Asia [43].

### Strengths and limitations

This study included a broad group of older aged patients form various towns and cities in Karnataka state and a diverse group of HCWs from several departments and different professions and took place for five months. Including older adults and HCWs and two qualitative research methods, resulted in triangulation of the data. However, this study has several limitations. The findings are limited by collection of the data in solely a teaching hospital and its urban health centre and by not including any governmental hospitals in Mysuru. However, including the urban health centre, which is located in the city’s outskirts, enabled the inclusion of the perspectives of a more varied patient population and their HCWs. Furthermore, that the interviews took place at the hospital might have led to socially desirable answers from the patients regarding their interaction with HCWs and their usage of preventive interventions. In addition, because the interviews took place in the working place of the HCWs, they might have given socially desirable answers regarding their interaction with the patients. However, the presence of SKS who is from Karnataka and who speaks Kannada and by performing hours of observations at several departments, often even before the interviews, might have helped to develop rapport.

## Conclusions

This qualitative study explored the perceptions of older adults regarding RTI prevention and the implications of the health care context in India with the aim to inform pharmaceutical and non-pharmaceutical prevention strategies for RTIs among older adults. The findings suggest a significant distinction between older adults’ attitudes towards and prevention efforts for COVID-19 and other respiratory infections. COVID-19 was seen as a severe disease which positively influenced uptake of vaccination, whereas seasonal respiratory infections were seen as inevitable, due to environmental factors, or were seen as preventable by a specific diet focused on either excluding ‘cold’ food and encouraging of ingesting ‘hot’ food. Therefore, highlighting potential risks of common respiratory infections and taking a holistic approach on prevention, which includes emphasizing a healthy diet during consultations by HCWs could be helpful. Furthermore, except for the COVID-19 vaccination, the concept of vaccination was not well understood among older adults. Language played an important role in effective health communication. Terms for influenza, common cold and vaccination in Kannada and English varied which could cause lack of understanding of adequate prevention of RTIs. Training on culturally appropriate communication for HCWs to enhance awareness of medical terms being used when informing older adults about prevention could be considered by hospitals. Finally, by including perceptions of seasonal respiratory infections and COVID-19, this research offers insights for (local) policy makers regarding effective preventive methods for endemic and emerging respiratory infectious diseases.

## Supporting information

S1 Appendix 30–60-minute in-depth semi-structured interview guide for older adults in Mysuru, India. (DOCX)

S2 Appendix 30–60-minute in-depth semi-structured interview guide for HCWs in Mysuru, India. (DOCX)

S3 Appendix COREQ (COnsolidated criteria for REporting Qualitative research) checklist. (PDF)
