# Supplementary material for: Perceptions of respiratory tract infections and their implications for disease prevention practices among older adults in Mysuru, India
Source: PLOS Glob Public Health. 2025 Jul 30;5(7):e0004982. doi: 10.1371/journal.pgph.0004982 (PMC12310005; doi:10.1371/journal.pgph.0004982)
Supplement: S1 Appendix — (DOCX) [file pgph.0004982.s001.docx]

## **S1 Appendix. 30-to-60-minute in-depth semi-structured interview guide older adults in Mysuru, India**

## **(Only includes the questions related to this article)**

| **Main themes** | **Questions English** |
| --- | --- |
| Demographic and background questions | In what year were you born?  In which neighbourhood do you live?  What are your living arrangements? How do you live?  Who else lives with you?  Do you work? Did you work?  How many years of education have you completed?  What is the highest level of education you have completed?  What made you come to the hospital? |
| Respiratory infections | Did you hear of any respiratory infections? Which ones? (common cold/flu/ *śītajvara* /H1N1/swine flu/ *handi jvara* /COVID-19/TB/pneumococcal disease)  What do you know about those?  Symptoms?  Do you see the flu as an important disease?   - Is the flu more or less severe/dangerous than COVID? - And common cold/H1N1?   Who do you think are at risk of getting infected with a respiratory infection? Or to get severe disease from an infection?  Have you ever had the flu?   - When was the last time you had the flu? - How did you feel when you had the flu? What kind of symptoms did you have? Were you worried?   Did you ever have any other respiratory infections?  How did you get these infections?  Did you get advice when you got sick? From whom?  How do you prevent getting sick from these infections? Did this change because of the COVID-19 pandemic? |
| Adult vaccines | Have you ever heard of vaccination/*lasike*?  Have you ever received a vaccine as an adult?   - Do you know about the flu vaccine/one-year-injection/pneumococcal vaccine/COVID-vaccine? - Which vaccine? - When was the last time you received this vaccination? - Since when did you receive this vaccine? - What motivated you to get vaccinated? (protecting yourself/others?) - Did you ever doubt to take this vaccine? How come?   If not: Did you ever adult vaccination in the past? What made you change your decision? Did this change because of the COVID-19 pandemic?  If not: What made you decide to not take the vaccine (this time)? Did this change because of the COVID-19 pandemic?  Could you describe your experience with the vaccine?  Could you describe your experience with the flu vaccine from last season? Was it different because of COVID?  How do you feel about receiving the flu vaccine each year?   - Can you give an example of/ could you tell me more about this negative/positive experience with the flu vaccine? - What would you like to change? |
| Individual and group influences | How do you prevent yourself from getting sick? / How do you stay healthy?  And when you are sick what do you do?  Does vaccination fit with this? And how do you protect others?  What do you know about the flu vaccine/covid vaccine/pneumococcal vaccine?  With whom do you talk about preventive methods and these vaccines?   - Can you tell me what you discuss with… during such a conversation? - What did you think of the conversation? Did you agree with them? Why (not)? - How did you feel afterwards? - Did this influence your decision to take the vaccine?   Did you get as a child all the vaccinations?  Would you recommend adult vaccines to your friends or family who are 60 years or older? What would you say to them? |
| Interaction with the health care worker | How do you experience your interactions with the doctor in the hospital?  Do you sometimes visit the GP/family doctor/doctor in the village? Do you talk to the GP sometimes?  How do you experience your interactions with the GP?  Do you sometimes talk to the doctor in the hospital or the GP about the flu vaccine/covid vaccine/pneumococcal vaccine/other measures against respiratory infection?   - What did you discuss? Can you give an example? - How did you feel during such a conversation? - Do you trust their recommendation? What makes you trust/distrust their recommendation? - What did you do with their advice?   With which other care takers did you talk about the flu vaccine?  If not: Can you tell me what made you not have this conversation with them? Were there other care takers with whom you talked about adult vaccines or other preventive measures? |
| Contextual influences | How did you become aware of the option to receive the flu/covid/pneumococcal vaccine? Who told you this?   - How do you get information about these vaccines/preventive measures? (community leader, government, (social) media, doctor, etc.) - Do you browse for information about respiratory infections, preventive measures/vaccination? What are you looking for? - What do you think of this information? Do you trust this information?   How do you feel about receiving an invitation for the influenza/covid/pneumococcal vaccine?  Where do you get your flu /covid/pneumococcal vaccine?  What do you think about the accessibility of the vaccine location?   - How do you reach the vaccine location? - What do you think about the location? Ambience? Personal attention? - Where would you prefer to receive these vaccines?   Do you have to sign up for the vaccination? How? What do you think about that?  What do you think of the costs of the vaccination?  Do you experience any barriers? |
| Vaccine/vaccination specific issues | What are the benefits of the flu/covid/pneumococcal vaccine for you?   - And for your environment? - And the risks?   What do you think of the effectiveness?  Do you trust the safety of the vaccine? What makes you trust or distrust the vaccine?  How do you feel about receiving vaccines via an injection with a needle?   - What makes you feel uncomfortable with needles? |
| COVID | Did the covid pandemic play a role in your decision to get the flu/pneumococcal vaccine? In what way? |
| Closing | Do you have any questions about the research?  Do you have any questions about the interview? |
