# Supplementary material for: Perceptions of respiratory tract infections and their implications for disease prevention practices among older adults in Mysuru, India
Source: PLOS Glob Public Health. 2025 Jul 30;5(7):e0004982. doi: 10.1371/journal.pgph.0004982 (PMC12310005; doi:10.1371/journal.pgph.0004982)
Supplement: S2 Appendix — (DOCX) [file pgph.0004982.s002.docx]

**S2 Appendix. 30-to-60-minute in-depth semi-structured interview guide for HCWs in Mysuru, India**

**(Only includes the questions related to this article)**

**Start recording**

| **Main themes** | **Questions English** |
| --- | --- |
| Demographic and background questions | In what year were you born?  In which department(s) do you work?  Can you tell me something about the work that you do?  For how long have you been doing this work?  What did you do before? |
| Respiratory infections | What do you know about respiratory infectious diseases?  What are important infectious diseases?  What do you know about influenza? How do you know about this?  Do you see influenza as an important disease?   - Is influenza more or less dangerous than COVID-19? - And for older adults?   Have you heard of pandemic influenza? How do you know about this?  Who do you think are more at risk to get infected? (respiratory infections) Or to get severe disease from an infection (respiratory infection)?  (How do you prevent yourself from getting sick/how do you stay healthy? Did this change since the COVID-19 pandemic? Does vaccination fit with this?) |
| Vaccination | What do you know about adult vaccinations? How do you know about this?  At what time of the year should adults get influenza/pneumococcal/COVID-19  vaccination? How do you know about this?    Which adult vaccinations are provided at the hospital? |
| Individual and group influences | What do your colleges think of adult vaccination (specifically for resp diseases)?  Do you agree with them? Why (not)?  Can you tell me what you discuss with your colleagues during such a conversation?   - What did you think of the conversation? - Did you agree with them? Why (not)?   Does your manager/head of department/supervisor ever talk about adult vaccination? What does he/she say? |
| Interaction with the health care worker | Do you talk about respiratory infections/non-pharmaceutical prevention methods to prevent respiratory infections /about how to treat respiratory infections?  with older adults?   - Who starts the conversation most of the time? - What did you discuss? Can you give an example? - How did you feel during such a conversation? - How did the patient act in such a conversation? - Is this common or do some patients act differently? - What motivated you to talk about this? Has it been advised by your manager/head of the department/hospital? - Are these conversations about how to avoid getting infected or how to avoid transmission or both?   Does the hospital where you work offer adult vaccinations to your patients who are 60 years and older? What is the reasoning behind this?  Do you talk with your patients who are 60 years and older about adult vaccination?   - What motivated you to talk about this? Has it been advised by your manager/head of the department/hospital? - Who starts the conversation most of the time? - What did you discuss? Can you give an example? - How did you feel during such a conversation? - How did the patient act in such a conversation? - Is this common or do some patients act differently? - Do patients refuse the vaccine? And why?   If not: Can you tell me what made you not have this conversation with patients aged 60 years and older? (Has this been advised by your manager/hospital?)  Does your own opinion regarding vaccinations influence the way you talk with your patients about adult vaccination/ non-pharmaceutical prevention? How?  Do you feel like you have enough knowledge to advice your patients about vaccination and non-pharmaceutical prevention methods against respiratory infections? Is this something you have learned during your studies or while working in the hospital?  Did the covid pandemic impact how you advise/educate your patients about respiratory infections and how? |
| Contextual influences | Do the patients get an invitation for the vaccination?  Which patients get an invitation?  Where do they get the vaccination?  What do you think of the accessibility of the location for older adults?  What are the costs of the vaccination?  Is that a problem for some patients, including older adults?  How do they reach the vaccination location?  Do they have to sign up for the vaccination? How? |
| Vaccination/vaccination specific issues | What are the benefits of the adult vaccination for older adults?   - And for their environment? - And the risks? - What makes you think this? Where did you get this information?   Do the patients trust vaccination?  What do they think of the effectiveness of adult vaccinations?  Did this change during the COVID-19 pandemic?  How do the older adult patients feel about receiving adult vaccination via an injection with a needle? |
| Closing | Do you have any questions about the research?  Do you have any questions about the interview? |
